# A Combined Experimental and Theoretical Study of Screen-printing High Transparent Conductive Mesoscopic ITO Films

**DOI:** 10.1038/s41598-020-61124-w

**Published:** 2020-03-19

**Authors:** Feiyang Qiao, Lei Lu, Pingcheng Han, Dekai Ge, Yunjun Rui, Dawei Gu, Tianyou Zhang, Jiwei Hou, Ying Yang

**Affiliations:** Department of Physics, School of Physical and Mathematical Sciences, Nanjing Tech University, NO.30 Puzhu Road, Nanjing, 211816 Jiangsu China

**Keywords:** Solar cells, Structural properties

## Abstract

We have successfully fabricated transparent conductive mesoporous indium tin oxide (TCM-ITO) films by a screen-printing method. The TCM-ITO films possess approximately 22 nm mesopores and obtain electrical conductivity up to 14.96 S/cm by adjusting the mass ratio of cubic-shaped ITO nanoparticles to ethyl cellulose (EC) and precisely controlling the annealing process. The regulation mechanism of EC and the heat-induced recrystallization process of ITO nanoparticles are elaborated. The internal kinetic processes of the films based on different surface states are analysed, and an extensible impedance model is established.

## Introduction

Transparent conducting oxides (TCOs) have been studied sufficiently in recent decades. Indium tin oxide (ITO) is widely used owing to its relatively high transparency, excellent conductivity, and suitable work function^1^. TCO films with mesoscopic structures have raised much attention in optoelectronics displays^2–4^, solar cells^5,6^ and sensors^7^. The combination between mesoscopic TCO and other photosensitive species can promote the separation of electrons and holes in the photosensitive materials, reduce the resistance during transmission, and effectively collect photoelectrons^8,9^. As an excellent TCO material, ITO films with mesoporous framework have raised significant attention because such a scaffold allows functionalization to obtain excellent device performance with electrochemical and photoelectrical active species^5,10^. Methods of controllable ITO mesoporous films have been realized by the sol-gel process^11^, but it yields amorphous pore walls and restricts the range of application on other mesoporous metal oxide substrates. Zhang *et al*.^12^ obtained the precursor solution with mixing tin chloride, indium chloride, and CTAB in ethanol, and deposited mesoporous ITO films on the quartz plates by dip coating. However, the flat substrate and the fluidity of the solution limit its range of application. At the same time, Michael gross *et al*.^13^ researched the mesoporous ITO films made by doctor blading ethanolic nanoparticles ITO dispersion. However, doctor blade method has some uncertainty and could not achieve precise control of film thickness. In view of that, a new method is needed to accurately prepare mesoporous films. Screen printing method, as a widely used method in fabricating films, could precisely control the thickness of the experimental film by adjusting various parameters. Whereas few efforts have been devoted to the systematic investigation of ITO mesoporous films manufactured by the screen printing method.

Hence, we proposed a series of strategies to fabricate robust ITO mesoporous films that have high conductivity and transmittance by using a paste consisting of pre-prepared ITO nanoparticles and the screen-printing technique, and that have mesopores of approximately 22 nm, electrical conductivity up to 7.6 S/cm, and average transmittance about 90% at 500 °C. The properties of mesoporous ITO films produced by different methods are listed in Table S1. Moreover, we have elaborated the regulation mechanism of EC, the process of heat-induced recrystallization of ITO nanoparticles, and the electrochemistry characteristics of ITO films as transparent electrodes. The internal kinetic behaviour of the films with different surface states is explored, and an extensible impedance model based on conductive mesoporous films is offered to estimate the electrode performance.

## Results and Discussion

In this work, pre-prepared ITO nanoparticles were applied as an obstacle to limit the pore size in the mesoporous scaffold. Typically, the intensive nucleation, growth, coagulation, and flocculation of ITO crystals limit the grain size and pore size in the sol-gel process^2^. Thus, by introducing pre-prepared ITO particles with a specific giant size, it is possible to obtain films with mesopores of the same scale. Moreover, several articles have reported that it is feasible to control the grain size and shape of the nanoparticles^14–16^, by which the size of the mesopores can be arbitrary regulated by adjusting the grain size.

**Figure 1:**
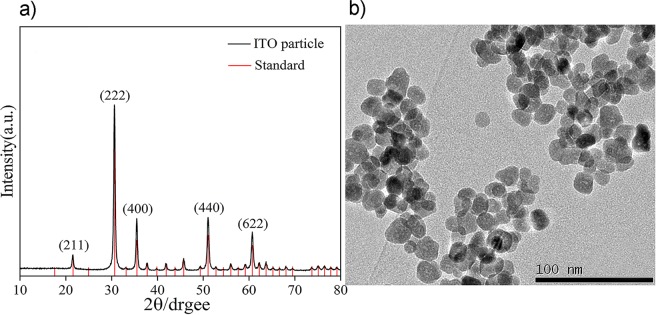
(**a**) X-ray diffraction (XRD) patterns of ITO nanoparticles and the standard patterns (**b**) Transmission electron microscopy image of ITO nanoparticles.

Under the conditions described above, we synthesized cubic-shaped ITO nanoparticles to fabricate ITO mesoporous films. The cubic particles have line-to-line and face-to-face contact among them, which enhanced the conductivity significantly compared with spherical ones^17^. By analyzing the X-ray diffraction (XRD) patterns of ITO nanoparticles in Fig. 1a, it can be found that the peaks at 21.497°, 30.585°, 35.462°, 51.024°, and 60.667° corresponding to the (211), (222), (400), (440), and (622) indices of crystal face of ICDD PDF#71-2194, which obviously indicate that the ITO nanocrystals have a simple cubic structure compared with the standard patterns.

Electronic conduction in pure indium oxide is caused by the oxygen vacancy, which can provide two electrons. In ITO, each donor ion Sn^4+^ substitutes for In^3+^ in the original lattice position, giving rise to an additional electron in the conduction band. Generally speaking, a greater number of Sn^4+^ ions doped in In_2_O_3_ promise a higher conductivity, whereas the cassiterite structure of the SnO_2_ phase is observed in the case of high Sn^4+^ content (>30%). Moreover, the crystallite size of the powder augments simultaneously with the increase in Sn^4+^ content^18,19^. Thus, to prevent the appearance of a second phase and control the grain size, the ratio of Sn^4+^ to In^3+^ in the synthesis is kept at 1:10.

Consistent with this, no phases corresponding to tin oxide are detected in XRD patterns, showing that Sn dissolves in the In_2_O_3_ lattices completely. Additionally, the average size of the ITO crystals is approximately 23 nm, which is calculated according to the Scherrer equation:

1 $$D=\frac{K\lambda }{Bcos\theta }$$

**Figure 2:**
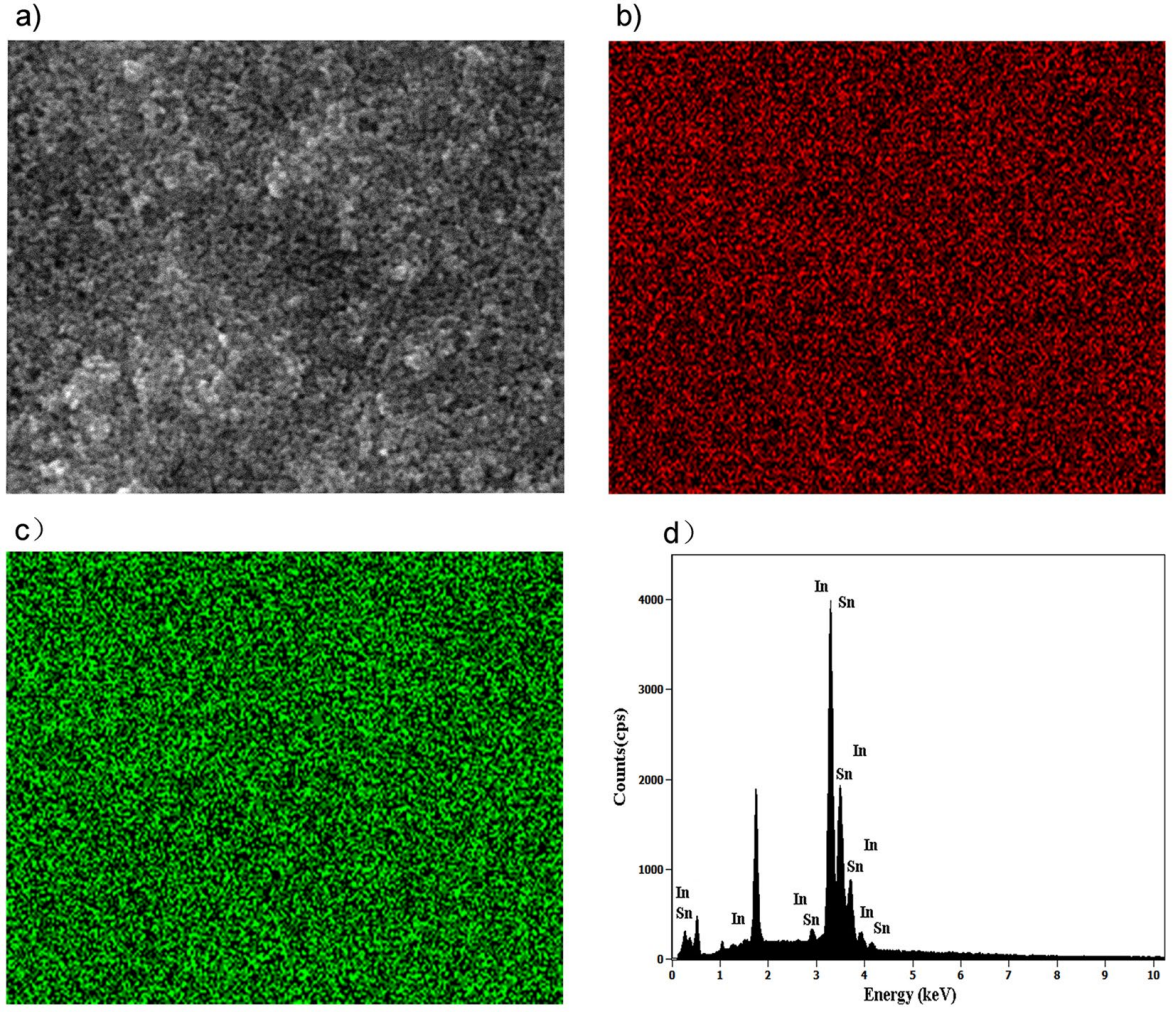
(**a**) Gray image of scanned microdomain (**b**) Distribution of In (**c**) Distribution of Sn (**d**) EDS spectra of the TCM-ITO film.

Such average grain size and crystal shape are confirmed by TEM analyses in Fig. 1b, which shows the presence of cubic nanocrystallites. Based on observations of the film morphology, the microdomain element distribution of the ITO film is analysed by an energy dispersive spectrometer to estimate the actual element ratio (Fig. 2).

According to Fig. 2d, X-ray emission is detected at 3.287 keV (*Lα*), 3.487 keV(*Lβ*_1_), 3.714 keV(*Lβ*_2_), 3.912 keV (*Lγ*_1_) for In, and 3.444 keV (*Lα*), 3.663 keV(*Lβ*_1_), 3.905 keV(*Lβ*_2_), 4.131 keV (*Lγ*_1_) for Sn^20^, with the element ratio of In to Sn is 1:10.57, which is close to the original ratio in the solvothermal reaction. Also, the EDS maps (Fig. 2b and Fig. 2c) exhibit an overlay of In and Sn with a complex mixture in the ITO films and provided evidence for the doping of tin in the In_2_O_3_ lattices.

Generally, sol-gel-derived mesoporous films employing block copolymer as templates result in some cracks and shrinking of the mesopores size by up to 50%, owing to the lack of adhesion^8^. Herein, we employed EC as a surfactant and binder to settle this problem.

**Figure 3:**
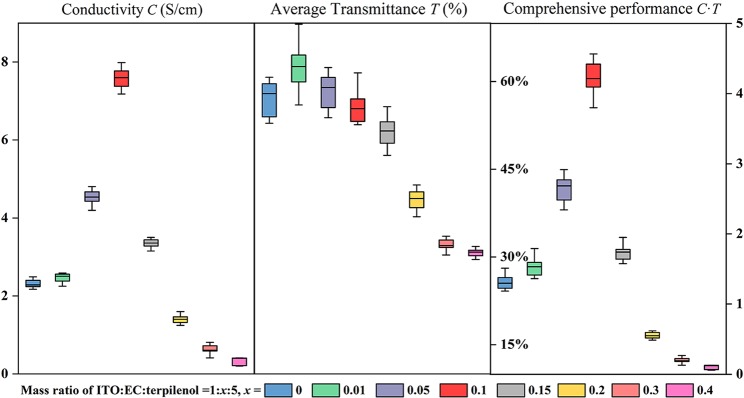
Box diagram of conductivity, average transmittance and comprehensive performance of TCM-ITO films with different doping amount of ethyl cellulose.

In the process of screen printing, several pastes with different mass ratios of ITO nanoparticles to EC were prepared. The modulation of mass ratio allows us to investigate the regulation mechanism of EC during the recrystallizing process and its impact on the optical and electrical properties of the films. Usually, more EC added leads to larger mesopores in the nanocrystals, and the sheet resistance or transmittance will be affected negatively, whereas the appropriate addition of EC can profoundly improve the conductivity, as shown in Fig. 3. The optimal resistance and transmittance are obtained through repetition, and each data is based on the average of twenty samples. That is where the regulation mechanism of EC reflected, and the film has optimal comprehensive performance when the ratio of m_EC_:m_ITO_ is 0.1.

The regulation mechanism of EC is inseparable from its chemical and thermodynamic properties. It has a self-assembly tendency to form regular micelles and prompt the nanoparticles into the mesoporous framework due to different hydrophilic properties of the radical group and hydrogen bond in it when the solvent evaporates at 70 °C^21^. With the temperature further increased to 300 °C, the ITO nanoparticles underwent thermal expansion during annealing, which caused the crystals to collide with each other and form cracks, thereby leading to parts of the conductive path to be interrupted. However, by virtue of the long molecular chain, the self-assembled micelle of EC buffered the expansion process and redistributed the internal thermal stress of ITO nanoparticles^22^. At this temperature, the ITO nanoparticles began to melt, expand, and recrystallize to form sizeable conductive channels. Thus, serving as a binder, the proper viscosity of EC also increased the connection among ITO crystals during the annealing process.

EC would not be burned off until the temperature of the mesoporous structure increased to 400 °C, which avoided the structural collapse. Afterward, the nanoparticles further melted and recrystallized during the process of heating to 500 °C, and EC had already decomposed thoroughly at 500 °C.

Experimentally, the properties of the films were found to have specific temperature dependence and ascertained as a heat-induced recrystallization process. In general, the films’ conductivity is derived from the conductive channels formed by recrystallization of the particles. The decomposition temperature of the surfactant and crystallization temperature of ITO should be strictly coordinated. Otherwise, the templates not completely decomposed during crystallization may cause a rise in impurity concentration inside the films. In that, the premature burn-off of the templates in the sol-gel process leads to thickening of pore walls, though promotes conductivity to some extent; however, it limits the film thickness or even results in structure collapse^8,23^. By precise controlling, the heat-induced recrystallization process happened above 300 °C, and at this temperature, EC still had a stable micellar structure, which ensured the shaping of large mesopores and uniform mesoporous structure.

**Figure 4:**
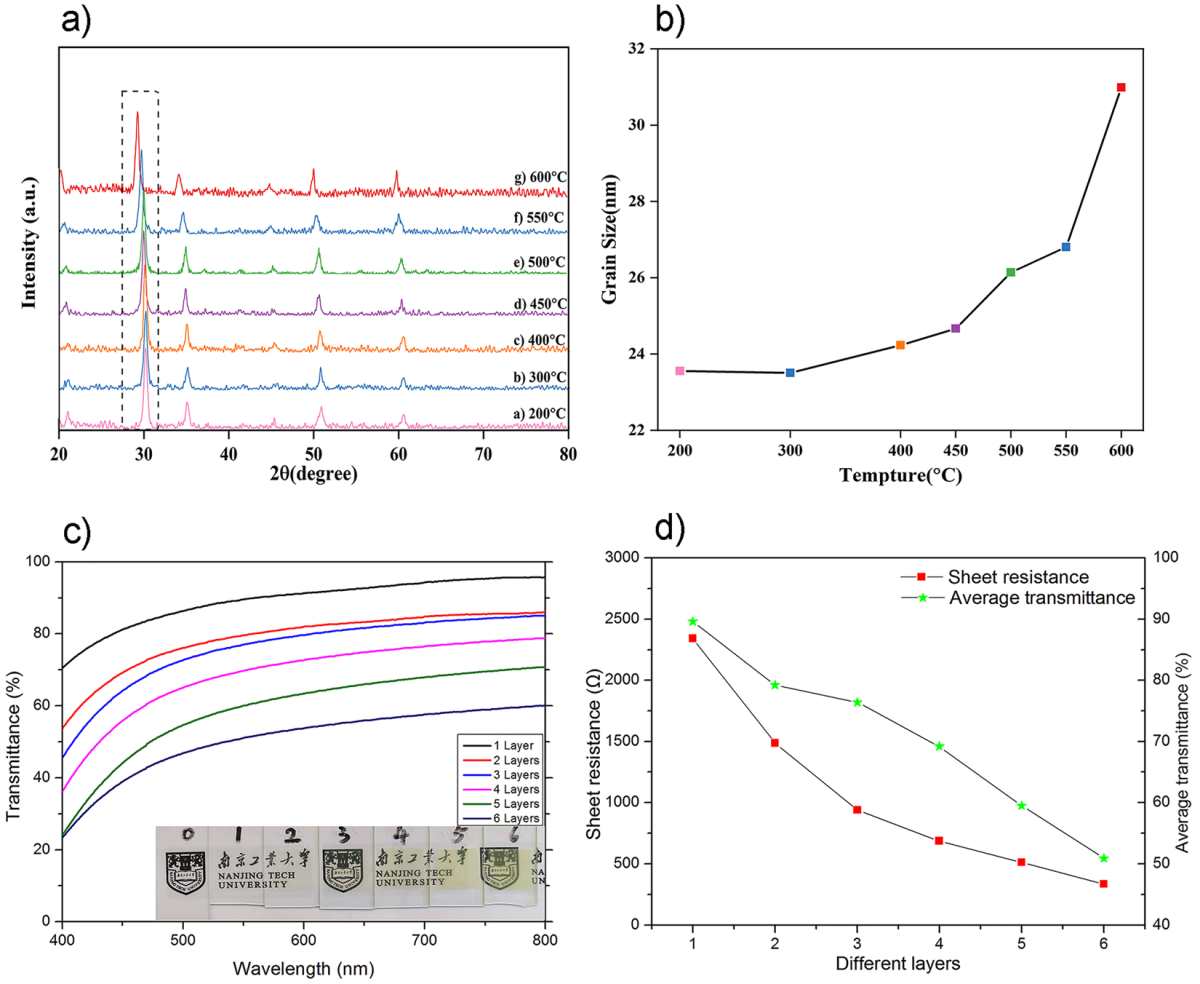
(**a**) XRD patterns of TCM-ITO films and (**b**) grain size of TCM-ITO films calcined at different temperatures, (**c**) the UV-vis transmittance spectra and (**d**) the sheet resistance and average transmittance of TCM-ITO films with different thickness.

The XRD patterns of films calcined at different temperatures were measured to prove the above discussion. As shown in Fig. 4a, the main peak exhibits a slight leftward shift and width expansion tendency, revealing the extension in the crystal face. To estimate this crystal extension, the average crystallite size was calculated according to the Scherrer equation, as shown in Fig. 4b. The crystal was tightly integrated and became larger during the heat-induced process. Thus, the crystal status significantly affected the films’ electrical properties. The conductivity of the TCM-ITO films at different temperatures is listed in Table 1.

**Table 1:** Properties of m-ITO films calcined at different temperatures.

Annealing Temperature (°C)	Sheet resistance(Ω/sq.)	Conductivity(S/cm)	Average transmittance (%)
200	98730	0.133	58.77%
300	78110	0.168	57.17%
400	7884	1.660	49.78%
450	5331	2.455	51.33%
500	2214	7.604	56.38%
550	1370	9.554	55.38%
600	875	14.959	61.64%

Evidently, in Table 1, as the temperature increases, the films show better electrical conductivity, also coinciding with the recrystallization hypothetical theory of oxygen vacancies redistribution^24^. Therefore, a high annealing temperature is beneficial for conductivity and when annealed at 600 °C, the film’s conductivity is up to 14.96 S/cm. However, the transmittance of the TCM-ITO films does not change significantly with increasing temperature. This shows that the TCM-ITO’s transmittance is less affected by temperature, so it can be used as transparent films in photovoltaic devices. For energy and application considerations, we applied the annealing temperature of 500 °C to reduce impacts on photovoltaic devices, such as solid-state solar cells.

ITO films prepared via other process are very thin (<100 nm) owing to insufficient adhesion during annealing or instrument limitation^8,11,12,25–27^. To lift this restriction, the screen-printing technique is employed to supplant dip-coating or spin-coating methods, which can form film precursors with suitable viscosity. Furthermore, the thickness of the films can be adjusted by changing the printing times to obtain high conductivity and considerable transmittance.

In order to improve the performance of the TCM-ITO film, we have ultrasonically treated the paste. In the work, we also researched the transmittance and conductivity of the TCM-ITO films based on different ultrasonic time. In order to observe the change of colour of TCM-ITO films more clearly, we prepared 6 layers TCM-ITO films based on 0.5 hour, 1.5 hours, and 4 hours, respectively by screen printing method, as shown in Fig. S1. It is clearly in Fig. S1 that the yellow colour of the TCM-ITO films with the same layers tends to become lighter as the ultrasonic time increases. Similarly, as the ultrasonic time was extended from 0.5 hour to 1.5 hours, the average transmittance of the TCM-ITO film increased to 49.5%. While, as the ultrasound time was further extended to 4 hours, the average transmittance of the TCM-ITO film became stable, and the film appeared pale yellow.

F-SEM patterns of TCM-ITO films based on different ultrasonic time were shown In Fig. S2. It is clearly that as the ultrasonic time increases from 0.5 hour to 4 hours, the agglomeration in the TCM-ITO film becomes less, and the surface of the TCM-ITO film becomes more uniform and flat. This result is consistent with the transmittance and the optical photographs of the TCM-ITO films based on different ultrasonic time in Fig. S1. Based on the above experimental result, it’s obviously that the transmittance of the TCM-ITO film will increase greatly when ultrasonic time extend, but when the processing time exceeds 1.5 hours, the ultrasonic treatment does not contribute much to the film transmission. Therefore, in the later experiments, we used a 1.5 hour ultrasonic ITO paste to prepare the TCM-ITO film.

The relationship between the performance of transmittance and conductivity under different layers of TCM-ITO films was researched in Fig. 4c and Fig. 4d. The transmittance of the TCM-ITO films increases as the wavelength increase from 400 nm to 800 nm, and the transmittance of single layer m-ITO film get a high transmittance above 95% at 800 nm and an average transmittance approximately 90% ranging from 400 nm to 800 nm. And as the layers increase, both the sheet resistance and average transmittance decrease.

Electrochemical impedance spectroscopy (EIS) is a generic technique that allows for the studying of electrical properties, such as transport, accumulation, transfer, and loss of charges in mesoporous electrodes. Impedance spectroscopy may be measured under operational conditions and can facilitate the analysis of electrical processes occurring in the system, enabling the development of models that describe the physicochemical behaviour of electrodes and, eventually, unveiling the mechanisms that enhance performance or produce degradation^28,29^. For these reasons, impedance spectroscopy is applied to characterize the electrochemical properties of the ITO electrodes and elucidate the effects of the mesoporous structure on electrode performance.

**Figure 5:**
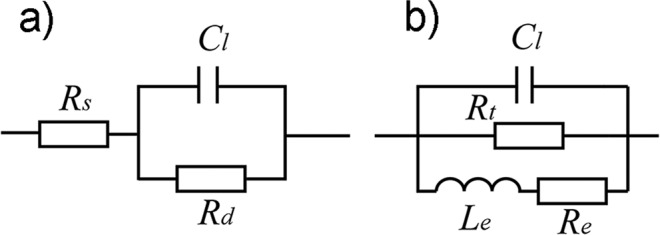
The equivalent circuit of (**a**) *R*_*s*_*(C*_*l*_*R*_*d*_*)* (**b**) *(C*_*l*_*R*_*t*_*(L*_*e*_*R*_*e*_*)*.

In typical electrochemical impedance spectroscopy analysis of mesoporous structures, several excellent reviews have ascertained that charge transfer mainly occurs at the grain and boundary in mesoporous materials, whereas the surface state is often systematically under estimated^30,31^. The equivalent circuit for a general mesoporous polycrystalline electrode is *R*_*s*_*(C*_*l*_*R*_*d*_), noted by Circuit Description Code (CDC) (Fig. 5a). Some researchers have noticed the dispersion effect of surface state in an experimental mesoporous electrode and proposed using constant phase element *Q* instead of capacitance *C*_*l*_ to fit the actual impedance spectra, noted as *R*_*s*_*(QR*_*d*_); however, only a simple dependence relationship was observed^32,33^.

Nonetheless, with the mesopores further enlarged and redox agents deposited, surface states of electrodes have an impact not only on the dispersion effect of the shallow surface but also on the internal kinetic and charge transfer steps^34,35^. Although the properties of ITO films under different atmospheres have been fully studied, seldom published is a systematic study concerning the theoretical impedance model analysis and detailed verification^36,37^.

To gain more insight on the complex kinetics of the electrode process, an oxidized In_2_O_3_ surface was taken as an example to illustrate the changes in impedance spectra because the oxygen vacancies tend to be oxidized when exposed to ambient atmosphere, which has a proximity internal process in a reductive atmosphere or active material^38,39^. Such an oxidation process harms the film’s resistance due to the limited conductivity of the O-vacancy-poor In_2_O_3_ surface, which have been investigated intensively and expressed in Kröger–Vink notation as^40,41^:

2 $${V}_{O}^{gg}+2e{\prime} +\frac{1}{2}{O}_{2}={O}_{O}^{x}$$

3 $${I}_{f}={I}_{+}-{I}_{-}$$

**Figure 6:**
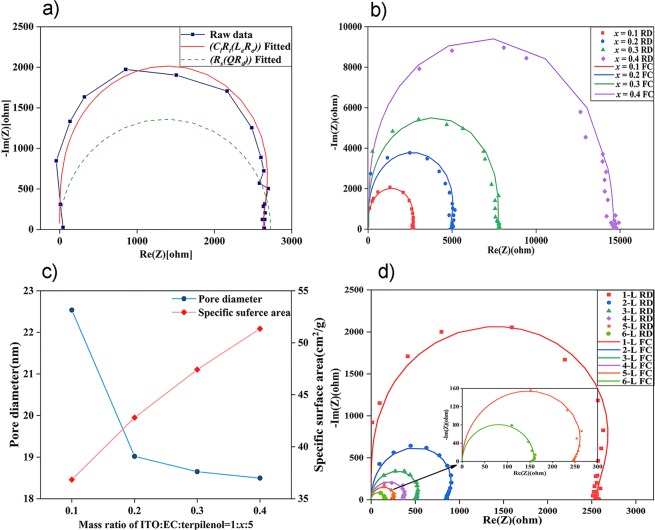
(**a**) Raw data (RD) of impedance spectroscopy and fitted curves (FC) based on different model (**b**) Impedance spectroscopy of films doped with different amount of EC (**c**) The specific surface areas and pore diameters of films doped with different amount of EC (**d**) Impedance spectroscopy of films with different stacked layers.

An impedance model of the mesoporous electrodes is established based on the electrode kinetic analysis and faradaic admittance model proposed by Martin Z. Bazant *et al*.^42–46^. For the sake of simplicity of the model, we controlled the annealing atmosphere of samples and aged them in air for 12 hours to gain a substantially equal thickness of O-vacancy-poor In_2_O_3_ film, but different coverage area *θ* due to separate specific surface area *s*. Commonly, a higher specific surface area promises a bigger coverage area due to more EC doped, which is proved in the BET measurement in Fig. 6c. The surface state variable *θ* was quantified to the coverage area of O-vacancy-poor In_2_O_3_ film and clearly, *θ* is proportional to *s*:

4 $$\theta \propto s$$

Therefore, *θ* is the only surface state variable when the diffusion effect caused by the concentration axial gradient is not considered^47^. The function *G* is introduced to indicate the rate of change of the coverage area *θ* with respect to time. It is clear that *G* is a function of *θ* and the electrode potential *E*^43^.

5 $$G=\frac{d\theta }{dt}=g(E,\theta )$$

Additionally, because the charge transfer process obeys Faraday’s law, the bulk resistance *R*_*d*_ should have the form of faradaic impedance *Z*_*d*_^43^:

6 $${Z}_{d}=\frac{1}{{Y}_{d}}=\frac{\Delta E}{\Delta {I}_{f}}$$

where

7 $${Y}_{d}=\frac{1}{{R}_{t}}+\frac{B}{a+j\omega }$$

8 $$B=m\cdot b={\left(\frac{\partial {I}_{f}}{\partial \theta }\right)}_{ss}\cdot {\left(\frac{\partial G}{\partial E}\right)}_{ss}$$

9 $$\frac{1}{{R}_{t}}={\left(\frac{\partial {I}_{f}}{\partial E}\right)}_{ss}$$

10 $$a=-{\left(\frac{\partial G}{\partial \theta }\right)}_{ss}$$

and the subscript ‘*ss*’ represents steady state.

According to the Butler-Volmer model, the positive and negative reaction current can be expressed as Eqs. (11) and (12) ^48,49^:

11 $${I}_{+}={k}_{+}(1-\theta ){C}_{{O}_{2}}exp\left(-\frac{\alpha FE}{RT}\right)$$

12 $${I}_{-}={k}_{-}\theta exp\left(\frac{(1-\alpha )FE}{RT}\right)$$

where *k*_+_ and *k*_*−*_ represent the standard rate constants of positive and negative reaction direction, respectively, *C*_O2_ means the external oxygen concentration, *α* represents the transfer coefficient, *F* is the Faraday constant, *R* is the gas constant, and *T* is the kelvin temperature. Moreover, we can obtain the reaction rate of the whole reversible reaction^50,51^:

13 $$G=\frac{d\theta }{dt}=K({I}_{+}-{I}_{-})$$

where *K* is the conversion coefficient to correct the experimental error in the Stern and Geary model. Therefore, by substituting Eqs. (11), (12), and (13) into Eq. (8), the analytical solution of *b* and *m* can be solved as

14 $$b={\left(\frac{\partial G}{\partial E}\right)}_{ss}=-\frac{KF}{RT}[\alpha {I}_{+}+(1-\alpha ){I}_{-}]$$

15 $$m={\left(\frac{\partial {I}_{f}}{\partial \theta }\right)}_{ss}=-\left(\frac{{I}_{+}}{1-\theta }+\frac{{I}_{-}}{\theta }\right)$$

In the equations, *α* is usually 0.5 when the faradaic current is not precisely measured^52^ and the other coefficients are all positive under the single-step charge transfer process. Then the sign of *b* and *m* can be figured out as $b < 0,$ $m < 0,\,B > 0$.

Then, *Z*_*d*_ can be reduced to the following expression:

16 $${Z}_{d}=\frac{1}{{Y}_{d}}=\frac{1}{\frac{1}{{R}_{t}}+\frac{1}{-\frac{|a|}{B}+j\omega \frac{1}{B}}}$$

From this formula, it can be found that under the condition of *B* > *0*, −*|a|/B* has the same dimension with a resistance (*Ω*·cm^2^), whereas *1/B* has the same dimension with an inductance (*H*·cm^2^). Thus, *Z*_*d*_ can be regarded as an equivalent circuit in which the equivalent resistance *R*_*e*_ and the equivalent inductance *L*_*e*_ are connected in series and then connected in parallel with the equivalent resistance *R*_*t*_. ^43^ Consequently, taking the original interface capacitance into account, the equivalent circuit can be modified to *(C*_*l*_*R*_*t*_*(L*_*e*_*R*_*e*_)*)*, as shown in Fig. 5b. Similarly, if the films were annealed in a reductive atmosphere (NH_3_ or H_2_), it can be discovered that *B* is negative, indicating the existence of a paralleled R-C equivalent circuit, as reported in many other works^53,54^. Simultaneously, it proves that the inductance or capacitance component in the analog circuit is induced by the surface states, rather than the ring current in the irregular mesoporous structure.

The theoretical impedance spectra of the mesoporous electrodes were fitted by least-squares approximation according to the equivalent circuit of *R*_*s*_*(C*_*l*_*R*_*d*_*)* and *(C*_*l*_*R*_*t*_*(L*_*e*_*R*_*e*_*))*.

The raw data and fitted curves are plotted in Fig. 6a. The tiny deviations between the raw data and the fitted curve confirmed the correctness of the theoretical model and indicated the impacts of the surface state in the electrode with a unique surface state. As shown in Fig. 6b, the electrochemical behaviour of the films doped with different amounts of EC was also investigated and fitted according to our model along with the fitting parameters listed in Table 2. Besides the excellent consistency, Fig. 6b reveals changes in semicircular diameters of the different films, which intrigued us to probe the causes and will be described below.

**Table 2:** Theoretical fitting parameters of the equivalent circuit.

1: *x* :5	*C*_*l*_	*R*_*t*_	*L*_*e*_	*R*_*e*_	_*τ2*_
1:0.1:5	4.981 × 10^−11^	1.807 × 10^13^	2.005 × 10^−4^	2633	1.311 × 10^−7^
1:0.2:5	4.661 × 10^−11^	2.609 × 10^13^	6.669 × 10^−4^	4897	2.282 × 10^−7^
1:0.3:5	4.233 × 10^−11^	1.116 × 10^12^	1.293 × 10^−3^	7725	3.270 × 10^−7^
1:0.4:5	3.788 × 10^−11^	3.644 × 10^13^	3.334 × 10^−3^	14570	5.519 × 10^−7^

To illuminate the changes in the experimental situation, we further qualitatively analysed the influence mechanism of surface state variable *θ*. From the above equations, we obtained the expression of equivalent resistance *R*_*e*_, *R*_*t*_, and equivalent inductance *L*_*e*_:

17 $${R}_{t}=\left(\frac{\partial E}{\partial {I}_{f}}\right)=-\frac{RT}{KF[\alpha {I}_{+}+(1-\alpha ){I}_{-}]}$$

18 $${R}_{e}=-\frac{|{\left(\frac{\partial G}{\partial \theta }\right)}_{ss}|}{{\left(\frac{\partial G}{\partial E}\right)}_{ss}{\left(\frac{\partial {I}_{f}}{\partial \theta }\right)}_{ss}}=-\frac{K}{{\left(\frac{\partial G}{\partial E}\right)}_{ss}}=-\frac{K}{m}$$

19 $${L}_{e}=\frac{1}{{\left(\frac{\partial G}{\partial E}\right)}_{ss}{\left(\frac{\partial {I}_{f}}{\partial \theta }\right)}_{ss}}=-\frac{RT}{mKF\left[\frac{1}{2}{k}_{+}{C}_{{O}_{2}}\exp \left(-\frac{FE}{2RT}\right)+c\theta \right]}$$

20 $$c=\frac{1}{2}{k}_{-}\exp \left(\frac{FE}{2RT}\right)-\frac{1}{2}{k}_{+}{C}_{{O}_{2}}\exp \left(-\frac{FE}{2RT}\right)$$

The positive direction is the main reaction direction, thus *k*_+_ largely outweighs *k*_*−*,_ while *m* and the constant coefficient *c* are negative and other parameters are positive or exponentially attenuated. Therefore, we concluded that *L*_*e*_ increases as *θ* increases, namely, in direct proportion. The specific surface areas and pore sizes of different films were measured and plotted in Fig. 6c. Theoretically and numerically, the statistical data manifested the qualitative proportional relationship between *s* and *L*_*e*_, coinciding with the fitting results in Table 2.

Moreover, the physical meanings of the parameters can be defined from the expression. *R*_*t*_ has the form of Faraday current as a function of electrode potential and occurs only on the O-vacancy-poor In_2_O_3_ surfaces. Therefore, *R*_*t*_ denotes the charge transfer resistance of the shallow surface. In the expression of *R*_*e*_, *θ* does not exist. Therefore, *R*_*e*_ indicates the charge transfer resistance among the grains. In the expression of *L*_*e*_, the Faraday current is a dependent variable, while electrode potential and *θ* are independent variables. This means that the change in the Faraday current depends not only on the change in the electrode potential but also on *θ*, which act in the same direction^42,55^. Therefore, the inductance component here can be deemed as the obstacle of surface impedance to charge transport and the effects of the surface electrochemical reaction.

We further estimated the electrochemical performance of films with the same specific surface area but different stacked layers (Fig. 6d). As analysed above, the intersection of the curves and X-axis is close to the sheet resistance value (Table 1) in a wide frequency range (100 HZ–100 KHz), which also shows extreme stability of the electrode system.

Furthermore, semicircular curves in the low-frequency region (<100 Hz) have some deformations. The intersections of the curves and X-axis show a shift to the origin side, especially in highly conductive films. To explain the anomalies in the experiment, the time constants of the reaction *τ*_1_ and the electrode transfer process *τ*_2_ are further explored:

21 $${\tau }_{1}=\frac{1}{a+{R}_{t}B}$$

22 $${\tau }_{2}={R}_{e}{C}_{l}$$

On the order of magnitude, the reaction time constant *τ*_1_ is close to the electrode transfer time constant *τ*_2_, resulting in the shrink of the inductive arc. Gradually, the inductive arc overlaps with the capacitive arc, constituting a semicircle with real parts contraction^28,34,46^. As a result, the contraction is more distinct as Re gets smaller. Also, this explains why there is no sense of an inductive arc in the fourth quadrant. Similarly, as the EC content increases, it can be seen from Table 2 that the transfer time constant time *τ*_2_ continuously increases, which also indicates that the ITO mesoporous film has worse conductivity.

In summary, the *(C*_*l*_*R*_*t*_ *(L*_*e*_*R*_*e*_)) equivalent circuit fits the experimental data well, and the theoretical kinetic analysis clarifies the electrochemical behaviour of mesoporous films based on different EC content. With the increase in EC content, both the capacitive reactance (*X*_*C*_*(Ω)* = *1/2πfC*) and the inductive reactance (*X*_*L*_*(Ω)* = *2πfL*) in the ITO mesoporous electrode increase. As a result, the equivalent resistance of the ITO mesoporous electrode increases, and the electrons transport time in the ITO mesoporous electrode increase. Because of its high specific surface areas and good electrical conductivity, the ITO mesoporous films will have a good application in the field of mesoscopic solar cells.

## Conclusions

Based on a solvothermal reaction, the cubic-shaped crystalline ITO nanoparticles were prepared to fabricate ITO mesoporous films, with EC as the surfactant. We obtained an optimal ITO mesoporous film with superior electrical conductivity and sizeable spectral transparency. Besides this, the mesostructure and film thickness can be controlled arbitrarily by screen-printing technology. The relevance of impedance spectroscopy and electrochemical properties were discussed, and an extensive impedance model was developed to fit and estimate impacts on the electrochemical behaviour of the electrode.

## Materials and Methods

### Synthesis of indium tin oxide nanoparticles

The nanoparticles were prepared by a solvothermal reaction in ethylene glycol (EG) according to the literature^14^. Typically, the indium chloride tetrahydrate (InCl_3_·4H_2_O, 99.99%, Sinopharm Chemical Reagent Co., Ltd) and the tin chloride pentahydrate (SnCl_4_·5H_2_O, 99%, Sinopharm Chemical Reagent Co., Ltd) were dissolved in EG and kept the concentration of In^3+^ and Sn^5+^ 0.5 mol/L and 0.05 mol/L respectively. Besides, an ethylene glycol solution of NaOH was prepared equivoluminally, and the concentration was 2 mol/L. All previous solutions were mixed with vigorous stirring until the mixed solution was clear, then which was transferred into a Teflon-lined stainless-steel autoclave of 50 ml. The autoclave was heated at 250 °C for 24 hours and then cooled to room temperature naturally. The product was washed by ethanol for three times after centrifuged at 4000 rpm for 10 minutes.

### Preparation of indium tin oxide paste and thin films

The ITO nanoparticles, EC (M9, Sinopharm Chemical Reagent Co., Ltd), and terpineol (99%, Shanghai Lingfeng Chemical Reagent Co., Ltd) were mixed at the mass ratio of 1:x:5 (x = 0, 0.01, 0.05, 0.1, 0.15, 0.2, 0.3, 0.4). The mixture was homogenized in a ball mill for 120 minutes. The TCM-ITO films were prepared by the size of 2 cm × 2 cm with screen-printing method. After dried at 70 °C, another stacked layer was printed on the substrate, and the samples were calcined to 500 °C by 5 °C per minute and kept for 60 minutes.

### Characterization and measurement

The X-ray diffraction patterns were recorded at a scan rate of 2°/minute on a DX-2700 type diffractometer with Cu-Kα radiation (λ = 0.15418 nm) under a voltage of 40 kV and a current of 30 mA. The ultraviolet-visible (UV-vis) transmittance spectra of TCM-ITO films were measured by Perkin-Elmer Lambda 50 spectrometer, and the average transmittance was calculated in the range of 400 nm to 800 nm. The sheet resistances of films were measured with four-point probes (ST2263, Suzhou Jingge Electronic Co. Ltd., Suzhou, China). The electrochemical impedance spectroscopy (EIS) measurement was taken on ZAHNER-electric (Gmbh & Co. KG., Germany) and measured from 0.1 Hz to 4 MHz with an amplitude of 5 mV. The morphology and microstructure of the films were scoped by scanning electron microscopy (SEM, JSM-6510, JEOL Ltd., Tokyo, Japan) along with the elemental analysis of the samples was conducted by Energy Dispersive Spectrometer (EDS, NS7, Thermo Fisher Scientific Inc., Waltham, MA, USA). And the high-resolution transmission electron microscopy (TEM) pattern was observed with a Tecnai G^2^–20 Twin (Thermo Fisher Scientific Inc., Waltham, MA, USA). The specific surface area was evaluated by BET and BJH method on BELSORP-max (MicrotracBEL Japan, Inc.).

## Supplementary information

Supplementary information

## Data Availability

The datasets generated and analysed during the current study are available from the corresponding author on reasonable request.
