# Supplementary material for: A Combined Experimental and Theoretical Study of Screen-printing High Transparent Conductive Mesoscopic ITO Films
Source: Sci Rep. 2020 Mar 19;10:5024. doi: 10.1038/s41598-020-61124-w (PMC7081275; doi:10.1038/s41598-020-61124-w)
Supplement: Supplementary file 1 — Supplementary information [file 41598_2020_61124_MOESM1_ESM.pdf]

Supplementary information for

A Combined Experimental and Theoretical Study of Screen-printing High Conductive Transparent ITO Mesoporous Films

Feiyang Qiao, Lei Lu, Pingcheng Han, Dekai Ge, Yunjun Rui, Dawei Gu, Tianyou Zhang, Jiwei Hou, Ying Yang*

Department of Physics, School of Physical and Mathematical Sciences, Nanjing Tech University, 30 Puzhu South Road, Pukou District, Nanjing 210009, Jiangsu, China

*Correspondingauthor:yingyang@njtech.edu.cn

Table S1 The properties of mesoporous ITO films prepared by different method

Deposition method	Film Thickness (nm)	Conductivity (S/cm)	Average transmittance (%)	Pore diameter (nm)	Specific surface area (m^2/g)
Screen printing	594	7.6	~90	22	53
Spin coating[S1]	2500	~0.6	~75	30	---
Dip Coating[S2, S3]	125-165	1.6-2.2	~80	35-45	50-60
Dip Coating[S4]	300	83.3	~80	2.36	329
Doctor blade[S5]	---	~7	~80	15-30	---
Sol-gel process[S6, S7]	---	3.7-9.5	~80	~13	~38.23

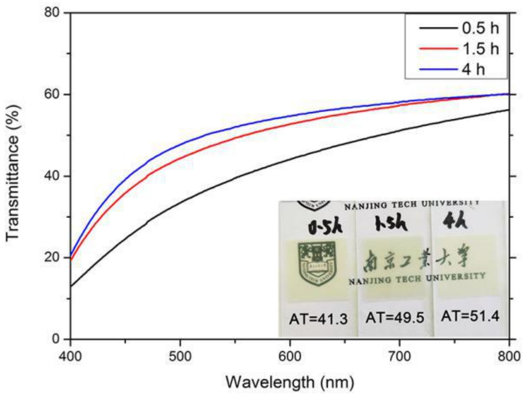

Figure S1. Transmittance of the TCM-ITO films of 6 layers based on different ultrasonic time

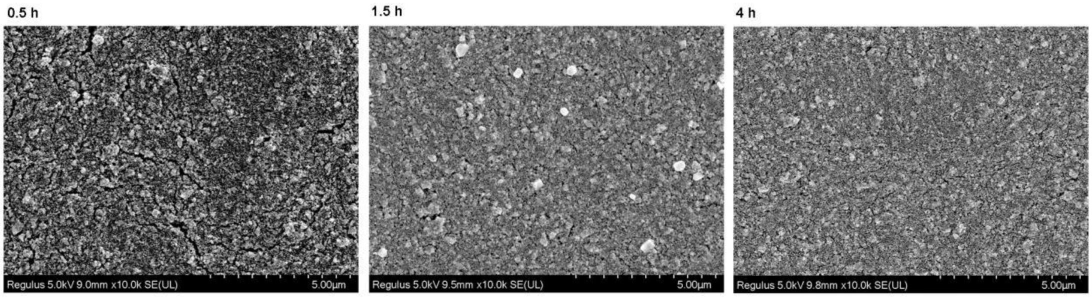

Figure S2. F-SEM patterns of the TCM-ITO films based on different ultrasonic time

References:

S1: Hoertz, P. G., Chen, Z., Kent, C. A. & Meyer, T. J., Inorganic Chemistry. 49(18), 8179-8181 (2010).

S2: Dunkel, C., von Graberg, T., Smarsly, B. M., Oekermann, T. & Wark, M., Materials. 7(4), 3291-3304 (2014).

S3: von Graberg, T. et al., Science and Technology of Advanced Materials. 12(2), 025005 (2011).

S4: Zhang, X., Wu, W., Tian, T., Man, Y. & Wang, J., Materials Research Bulletin. 43(4), 1016-1022 (2008).

S5: Gross, M., Winnacker, A. & Wellmann, P. J., Thin Solid Films. 515(24), 8567-8572 (2007).

S6: Daoudi, K. et al., Thin Solid Films. 445(1), 20-25 (2003).

S7: Liu, Y. et al., Chemical Science. 3(7), 2367-2374 (2012).
